# Repositionable Compounds with Antifungal Activity against Multidrug Resistant *Candida auris* Identified in the Medicines for Malaria Venture’s Pathogen Box

**DOI:** 10.3390/jof5040092

**Published:** 2019-10-01

**Authors:** Gina Wall, Natalia Herrera, José L. Lopez-Ribot

**Affiliations:** Department of Biology and South Texas Center for Emerging Infectious Diseases, The University of Texas at San Antonio, San Antonio, TX 78249, USA; gina.wall@utsa.edu (G.W.); n-herrera@live.com (N.H.)

**Keywords:** *Candida auris*, antifungals, drug screening, Pathogen Box, repurposing

## Abstract

Background. *Candida auris* has spread rapidly around the world as a causative agent of invasive candidiasis in health care facilities and there is an urgent need to find new options for treating this emerging, often multidrug-resistant pathogen. Methods. We screened the Pathogen Box^®^ chemical library for inhibitors of *C. auris* strain 0390, both under planktonic and biofilm growing conditions. Results. The primary screen identified 12 compounds that inhibited at least 60% of biofilm formation or planktonic growth. After confirmatory dose-response assays, iodoquinol and miltefosine were selected as the two main leading repositionable compounds. Iodoquinol displayed potent in vitro inhibitory activity against planktonic *C. auris* but showed negligible inhibitory activity against biofilms; whereas miltefosine was able to inhibit the growth of *C. auris* under both planktonic and biofilm-growing conditions. Subsequent experiments confirmed their activity against nine other strains *C. auris* clinical isolates, irrespective of their susceptibility profiles against conventional antifungals. We extended our studies further to seven different species of *Candida*, also with similar findings. Conclusion. Both drugs possess broad spectrum of activity against *Candida* spp., including multiple strains of the emergent *C. auris*, and may constitute promising repositionable options for the development of novel therapeutics for the treatment of candidiasis.

## 1. Introduction

Candidiasis is the third to fourth most common cause of nosocomial bloodstream infections in hospitalized patients in the United States [1,2,3,4], and invasive candidiasis carries mortality rates close to 40% [5]. Due to its prevalence and high morbidity and mortality rate, this infection presents a great challenge to clinicians. Even though *Candida albicans* is the main causative agent for these infections, infections caused by non-*albicans Candida* species (NACS) have increased in the last few decades, currently accounting for approximately half of the cases [6]. The most recent NACS to emerge as a formidable opportunistic pathogen is *Candida auris*. It was first reported in a human ear infection in 2009 in Japan [7], although it has been determined retroactively that it was present in an infection in South Korea in 1996 [8]. After its first identification, *C. auris* has since emerged throughout the world and become a major threat causing outbreaks of infections in hospitals and health care facilities worldwide [9,10,11,12,13,14,15,16,17]. There are several reasons that have contributed to the rapid spread of *C. auris*. First, unlike its distant relative *C. albicans*, *C. auris* is able to live on surfaces outside the human body for many weeks and remain on human skin for extended periods of time even after treatment, further complicating the management of these fungal infections [18,19,20,21,22,23]. For example, in New York City from 2013–2017, an outbreak of *C. auris* was monitored, and colonization was identified frequently as well as environmental contamination [24]. In the same outbreak, the mortality rate was 45% with 98% of cases having resistance to fluconazole. The second contributing factor is the difficulty in correctly identifying *C. auris*, which in the past had often been misidentified by commercial systems as other close species (i.e., *C. haemulonii*), possibly causing incorrect treatment regimens to be instituted thereby allowing the infection to persist [25,26]. The frequent antifungal resistance seen in *C. auris* represents a third contributing factor to its emergence and high mortality rates [27]. For example, it has been reported by the Centers for Disease Control and Prevention that 90% of *C. auris* strains in the U.S. have been resistant to fluconazole, 30% have been resistant to amphotericin B, and 5% have been resistant to echinocandins [28]. Thus, the fact that even some strains of *C. auris* can be resistant to all three major classes of clinically-used antifungals agents is a cause of great concern to the healthcare community [29,30]. Finally, *C. auris* has been shown to have the ability to form highly resistant biofilms, which allows for better defense against antifungals as well as immune cells [31,32,33]. 

In order to combat the increasing emergence of this fungal pathogen, it is imperative that new treatments be found. As opposed to a lengthy and expensive de novo pathway for drug discovery, repurposing or repositioning of existing drugs may represent a cost effective and faster approach to finding compounds with antifungal properties that can be readily used in patients [34,35,36,37]. To this end, here we screened The Pathogen Box^®^ library from Medicines for Malaria Venture (MMV; https://www.mmv.org/mmv-open/pathogen-box), a diverse library of approximately 400 drug-like compounds assembled by MMV, in search for inhibitors of *C. auris*. This library is made up of drug-like molecules that have been used to treat neglected tropical diseases like cryptosporidiosis, tuberculosis, malaria, and dengue, among other diseases, as well as reference compounds including some known antifungals [38]. Since the molecules in this library are known to possess activity in human infections, it is expected that the compounds identified in this screen could represent valuable options for the fast deployment of novel treatments against the devastating infections caused by *C. auris*, which are urgently needed.

## 2. Materials and Methods

### 2.1. Drug Library

The Pathogen Box^®^ was kindly provided by Medicines for Malaria Venture (Geneva, Switzerland). It contains 400 drug-like molecules, including reference compounds, which are known to be active against neglected diseases such as malaria, toxoplasmosis, and tuberculosis [38]. Two clinically-used antifungals, Amphotericin B and posaconazole, are also included. The molecules are provided in 96-well microtiter plates as 10 mM solutions in dimethyl sulfoxide (DMSO). From the main library, a daughter plate was prepared by making 1:100 dilutions and stored for future experiments at −20 °C. To make these solutions, 2 μL of each molecule was diluted into 198 μL of RPMI medium supplemented with L-glutamine (Cellgro, Manassas, VA, USA) and buffered with 165 mM morpholinepropanesulfonic acid ((MOPS) Thermo-Fisher Scientific, Waltham, MA) at pH 6.9, using the wells of presterilized, polystyrene, flat-bottomed 96-well microtiter plates (Corning Incorporated, Corning, NY, USA).

### 2.2. Strains and Culture Conditions

The *C. auris* panel was provided by the U.S. Centers for Disease Control and Prevention [39]. From this panel, *C. auris* 0390 clinical isolate was chosen for initial experiments, including the primary screens, because of its designation as multidrug resistant. This clinical isolate, classified as belonging to the South Asia clade I, was found to be resistant to azoles and amphotericin B and to have decreased susceptibility against echinocandins, according to the CDC. Eleven other clinical isolates, including nine other *C. auris* isolates, one *C. krusei*, and one *C. lusitaniae* from the CDC panel, as well as the *C. albicans* SC5314 type strain, and clinical isolates representative of different NACS obtained from the Fungus Testing Laboratory at the University of Texas Health Sciences Center at San Antonio, including *C. dubliniensis*, *C. parapsilosis*, *C. tropicalis*, and *C. glabrata*, were used in follow-up experiments.

The strains were grown overnight by inoculating cells in 20 mL of yeast extract-peptone-dextrose (YPD) (1% (*wt/vol*) yeast extract, 2% (*wt/vol*) peptone, 2% (*wt/vol*) dextrose) liquid medium in 150-mL flasks and incubating in an orbital shaker (150–180 rpm) at 30 °C. After 18–20 h, the cells were washed with phosphate-buffered saline (PBS) and counted with a hemocytometer. The cells were then diluted to the desired final density (usually 0.5 × 10^3^ cells/mL for planktonic testing and 1 × 10^6^ for biofilm testing) in RPMI. 

### 2.3. Primary Screens for Inhibitors of C. auris

The initial screens of the Pathogen Box^®^ for antifungal activity against *C. auris* were performed under two different growing conditions, planktonic and biofilm, using two different concentrations (5 and 20 μM) for each condition. To test the inhibitory effect of the molecules against planktonic *C. auris* 0390, the 5 and 20 μM screens were performed according to the CLSI document M27-A3 for antifungal susceptibility testing of yeasts [40], with minor modifications. Briefly, an inoculum of *C. auris* strain 0390 was prepared at 1 × 10^3^ cells/mL of yeast cells and added to the wells of 96-well microtiter plates, each containing an individual compound from the Pathogen Box^®^ at final concentrations of 5 or 20 μM. The plates were then incubated at 37 °C for 48 h, read visually (for >50% inhibition) at 24 and 48 h. At the end of the incubation, the cells in the wells were homogenized and the absorbance determined spectrophotometrically with a microtiter plate reader to provide a more quantitative measure of inhibition. 

To test the inhibitory effect of the molecules in inhibition of biofilm formation of *C. auris* 0390, the primary screenings (at 5 and 20 μM) were performed according to the 96-well microtiter plate model of *Candida* biofilm formation previously developed by our group [41,42]. Briefly, an inoculum of *C. auris* strain 0390 was prepared at 2 × 10^6^ cells/mL of yeast cells, and appropriate volumes were added to wells of a flat-bottom 96-well microtiter plate containing the same volume of compounds at appropriate concentrations, so that the final concentration of cells was 1 × 10^6^ cells/mL. The plates were then incubated at 37 °C for 24 h. Once incubation had ended, the plates were washed once with PBS to remove non-adherent cells, and the biofilm inhibition was estimated using a colorimetric assay on the basis of the reduction of 2,3-bis(2-methoxy-4-nitro-5-sulfo-phenyl)-2H-tetrazolium-5-carboxanilide (XTT, Sigma, St. Louis, MO, USA) over the course of 2 h by metabolically active cells as previously described by us [43]. 

For both screens, the first column (top four wells) as well as the last column (bottom four wells) served as positive controls (no compound added), while the first column (bottom four wells) and last column (top four wells) served as negative controls (no cells added), respectively. The screenings were performed at both 5 and 20 μM as mentioned above. Molecules found to inhibit 60% or more growth at 24 or 48 h in either screen (based on absorbance readings) were initially selected as “hits”.

### 2.4. Dose-Response Assays for Confirmation of Initial Hits

The confirmation of the activity of the compounds found to inhibit *C. auris* growth in the initial screens was accomplished through dose-response assays using the same microdilution techniques for inhibition of planktonic and biofilm growth as above. The starting concentration of the hits was 20 μM in both dose response assays, and serial 2-fold dilutions were done across the rows of a 96-well microtiter plate from left to right down to 0.0391 μM. Positive and negative controls were also included. To prepare the corresponding dose-response curve in both assays, the readings obtained from the plate reader were normalized using the positive (untreated) and negative (uninoculated) controls which were arbitrarily set as 100% and 0% growth. After performing these calculations, the IC_50_ values, defined as the concentration of drug required to reduce either planktonic or biofilm growth by 50%, were determined by fitting the normalized results to the variable slope Hill equation (an equation that determines the nonlinear drug dose-response relationship) using Prism 8 (GraphPad Software Inc., San Diego, CA, USA). 

### 2.5. Activity of Resupplied Miltefosine and Iodoquinol against C. auris 0390

For follow-up experiments, pharmaceutical grade iodoquinol and miltefosine were commercially purchased from Sigma-Aldrich, after identifying these two compounds as the most promising hits from the initial screens based on dose-response assays. For this set of experiments we tested the activity of these two drugs under three different modalities: planktonic growth and inhibition of biofilm formation (as described above), and activity against preformed biofilms, also as previously described by our group [41,42]. Briefly, *C. auris* 0390 was added to wells of a 96-well microtiter plate at a final concentration of 1 × 10^6^ cells/mL and incubated for 24 h to allow for biofilm formation. Once mature biofilms were formed, they were washed, and serial-dilutions of iodoquinol or miltefosine were added. The starting concentration for all three assays was 64 μg/mL for both drugs. The plates were read using the microtiter plate reader, with both biofilm assays being read colorimetrically using the XTT assay and the planktonic assay being read as absorbance to determine the turbidity of the wells after homogenization. As described for the dose-response assay, the readings were normalized and then the IC_50_ values determined using Prism.

### 2.6. Determination of the Activity of Miltefosine and Iodoquinol against Multiple C. auris Clinical Isolates and against Representative Strains of Different *Candida* Species

Both compounds were also tested against nine other *C. auris* clinical isolates in the CDC panel, *C. krusei*, and *C. lusitaniae* (also from the CDC panel), as well as *C. albicans* SC5314 type strain and representative isolates of *C. dubliniensis*, *C. tropicalis*, *C. parapsilosis*, *C. glabrata*. We used the same three in vitro assays (planktonic, inhibition of biofilm formation and activity against preformed biofilms) as described above. 

## 3. Results

### 3.1. Screening the Pathogen Box^®^ for Inhibitors of C. auris Planktonic Growth and Biofilm Formation, and Dose-Response Assays to Confirm the Activity of Initial Hits

We used two different 96-well microtiter plate-based models to perform initial screens of the Pathogen Box^®^ in search for compounds with inhibitory activity against *C. auris* under two different conditions: inhibition of planktonic growth (following CLSI methodologies, [40]) and inhibition of biofilm formation (using the model originally developed in our laboratory, [41,42]). *C. auris* strain 0390, from the CDC panel [39], was selected for these initial screens because it shows resistance to fluconazole and amphotericin B, and also displays decreased susceptibility against echinocandins according to the CDC. The screens were performed at two different concentrations under each growth condition, 5 and 20 μM. Compounds in the library were identified as initial hits if they inhibited growth in either assay by 60% or greater, based on spectrophotometric (for planktonic) or colorimetric (for biofilm) readings, as compared to the uninhibited controls. 

**Figure 1:**
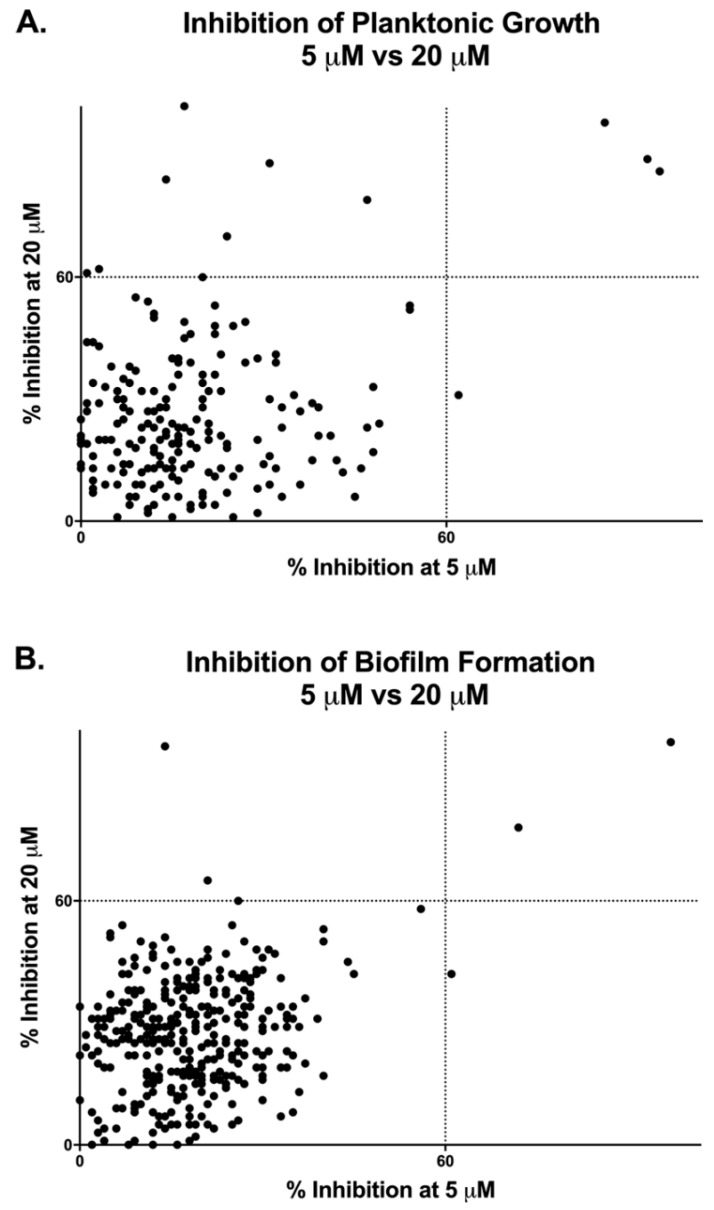
Graphical representation of results from primary screenings of the Pathogen Box^®^ in search for compounds with inhibitory activity against *Candida auris* strain 0390 under planktonic (**A**) and biofilm (**B**) growing conditions.

As shown in Figure 1 and Table 1, according to this criteria, we found a total of 11 compounds that inhibited *C. auris* planktonic growth at 20 μM, whereas only four of them were considered hits when screened at 5 μM. Likewise, a total of three compounds in the Pathogen Box^®^ library were capable of inhibiting *C. auris* biofilm formation by 60% or more at 20 μM, with only two of those representing hits when the screen was conducted at the lower concentration, though one other compound not identified at 20 μM was found at this concentration. As could be expected, all initial hits for biofilm formation overlapped with those capable of inhibiting planktonic growth. Overall, of the 400 compounds in the Pathogen Box, a total of 12 compounds fulfilled the criteria established for consideration as initial hits, resulting in a hit rate of 3%. Not surprisingly, some of the best hits were the fully established, Food and Drug Administration (FDA)-approved, and clinically-used amphotericin B and posaconazole, used in this collection as the two reference antifungal drugs, which served as internal controls for validation of screening results; however, we decided to exclude them from any further testing since our main interest was in finding repositionable compounds.

Next, we performed dose-response assays in order to provide confirmation of the activity of the remaining 10 compounds identified as initial hits during the primary screenings, and at the same time establish their potency. These assays were also done under both growth modalities (inhibition of planktonic growth and inhibition of biofilm formation), using the same microdilution methods as above, with the highest concentration tested being 20 μM. 

**Figure 2:**
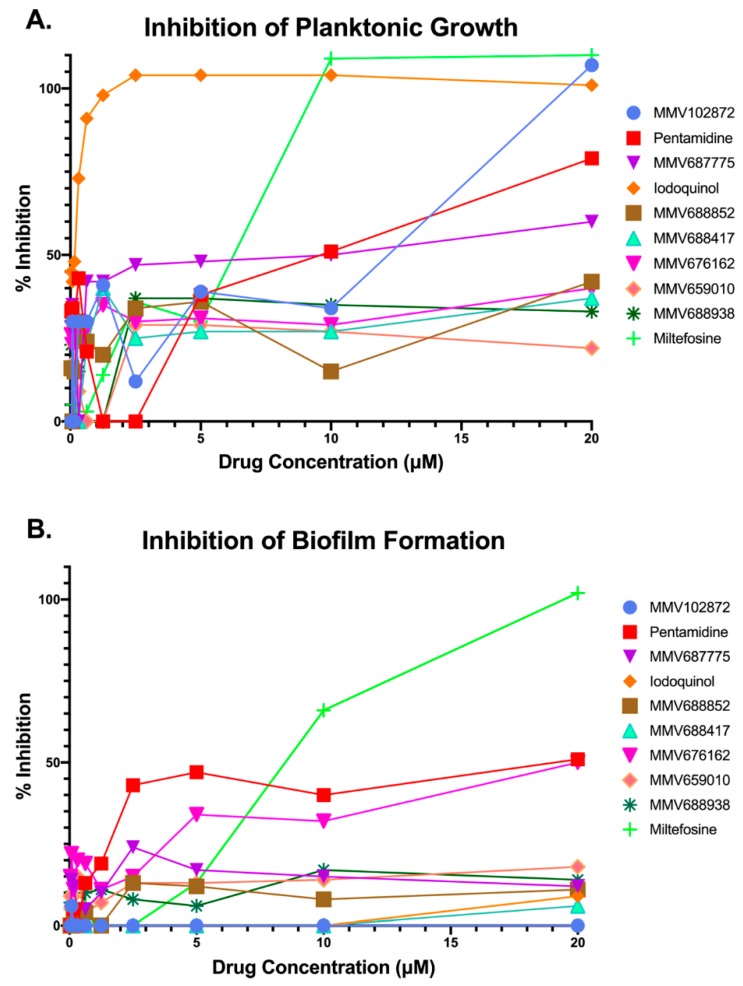
Results from dose-response experiments to confirm the inhibitory activity and determine the potency of initial hit compounds from primary screening against *C. auris* strain 0390 under planktonic (**A**) and biofilm (**B**) growing conditions.

Results confirmed the activity of four of the original 10 hit compounds (Figure 2). These four confirmed compounds were pentamidine, MMV687775, miltefosine, and iodoquinol. Although a nice dose-response was obtained for pentamidine, its potency against planktonic *C. auris* was significantly lower than the other three confirmed hit compounds (Figure 2A), and under biofilm growing conditions it did not achieve a 50% inhibition even at the highest concentration tested (Figure 2B). Similar observations were made for compound MMV687775 under planktonic conditions, barely reaching 60% inhibition even at the highest concentration tested (Figure 2A), and this compound was totally ineffective at inhibiting biofilm formation (Figure 2B). Iodoquinol displayed extremely potent activity against *C. auris* planktonic growth, with over 80% inhibition detected even at some of the lowest concentrations tested (Figure 2A); however, it was mostly ineffective at inhibiting biofilm formation (Figure 2B). Relatively similar dose-response curves were obtained for miltefosine under planktonic and biofilm growing conditions (Figure 2A,B), as treatment with this compound led to almost complete inhibition under both growth modalities at the highest concentrations tested. Based on these results and their repositioning potential, we chose to focus on miltefosine and iodoquinol for further characterization of their activity against *C. auris*. 

### 3.2. Follow-Up Studies with Miltefosine and Iodoquinol to Ascertain Their Inhibitory Activity against C. auris

**Figure 3:**
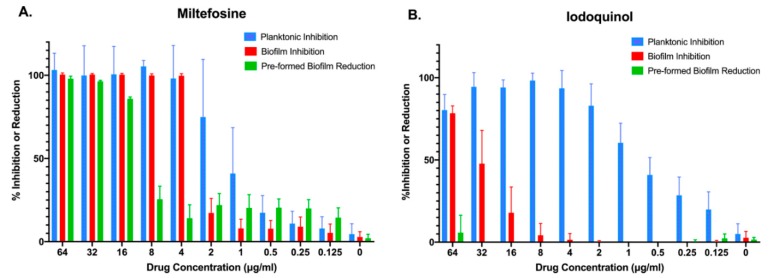
Activity of the two main repositionable compounds miltefosine and iodoquinol against *C. auris* strain 0390, under the three different growing conditions: inhibition of planktonic growth (blue bars), inhibition of biofilm formation (red bars), and activity against preformed biofilms (green bars).

Both miltefosine and iodoquinol are available from different commercial sources, and we purchased these compounds in order to ascertain their activity against *C. auris* and to secure enough quantities for additional follow-up studies. We performed similar dose-response experiments with the newly commercially purchased, pharmaceutical grade compounds in order to determine their inhibitory effect against the same strain of *C. auris*, under the same two different growing conditions (planktonic and inhibition of biofilm formation), and also extended these observations to a third treatment modality (activity against preformed biofilms). As seen in Figure 3A, and confirming our previous results, miltefosine was found to completely abolish planktonic growth and biofilm formation at relatively low concentrations (4 μg/mL), with over 60% inhibition of planktonic growth observed at 2 μg/mL. At 16 μg/mL, miltefosine was able to reduce close to 90% of metabolic activity of a preformed *C. auris* biofilm. Iodoquinol, on the other hand, was found mostly ineffective against *C. auris* biofilms, displaying virtually no activity against pre-formed biofilms and less than 80% inhibition of biofilm formation even at the highest concentration tested (64 μg/mL). On the other hand, it showed very potent activity against *C. auris* planktonic growth, with over 60% inhibition at concentrations as low as 1 μg/mL, and almost complete inhibition of proliferation at concentrations of 4 μg/mL and higher (Figure 3B).

We then extended our observations and examined the activity of miltefosine and iodoquinol against all other nine *C. auris* strains in the CDC panel (numbered 0381–0389, with different origins and patterns of susceptibility against conventional antifungals), also under the same three different treatment modalities: planktonic, inhibition of biofilm and activity against preformed biofilms. From the resulting dose-response curves, we calculated the corresponding IC_50_ values (the concentration of drug required to reduce growth of each strain by half). 

As shown in Table 2, results of susceptibility testing obtained for the different strains tested were mostly comparable to those previously observed for *C. auris* strain 0390. Iodoquinol displayed potent activity against planktonic growth of all strains tested, with IC_50_ values generally lower than 1 μg/mL; with slightly elevated values around 2 μg/mL detected for strains 0383, 0384, and 0385. Similar IC_50_ values, ranging from 1 to 2 μg/mL for all strains in the CDC panel, were calculated for planktonic growth in the case of miltefosine. Confirming our previous results, iodoquinol displayed rather poor anti-biofilm activity, with IC_50_ values for inhibition of biofilm formation generally over 20 μg/mL, and little to no activity against preformed biofilms of all different *C. auris* strains tested. On the other hand, miltefosine exhibited excellent biofilm-inhibitory activity against all strains tested, with IC_50_ values similar or only about one tube dilution higher (ranging from 1 to 6 μg/mL) to those that inhibited planktonic growth. Notably, relatively low IC_50_ values ranging from 9 to 15 μg/mL were calculated when the activity of miltefosine against preformed biofilms formed by the different *C. auris* strains was evaluated, with the exception of strain 0388, for which the drug showed a slightly elevated IC_50_ of 20.98 μg/mL. 

### 3.3. Activity of Miltefosine and Iodoquinol against Multiple Candida Species

**Figure 4:**
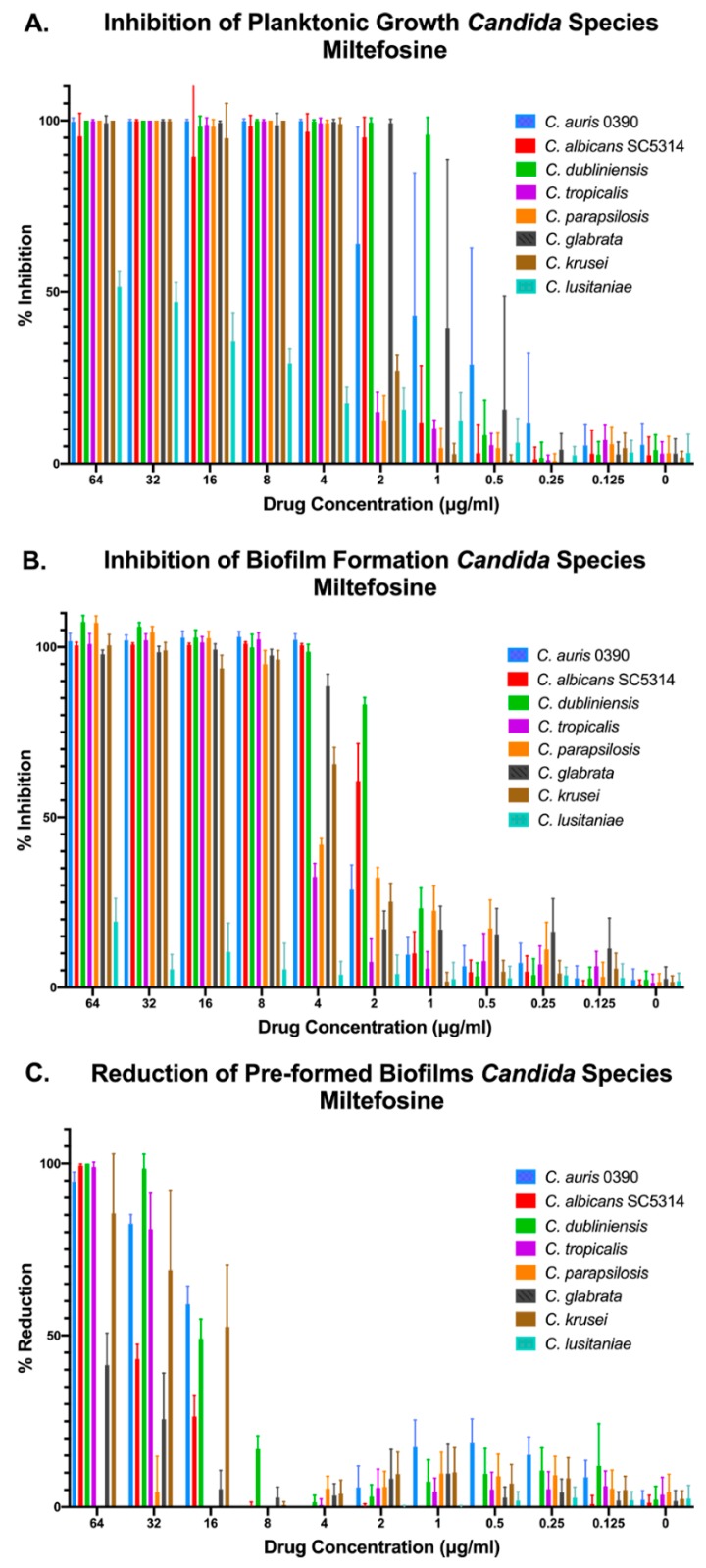
Activity of miltefosine against representative strains from different *Candida* spp. under the three different growing conditions: inhibition of planktonic growth (**A**), inhibition of biofilm formation (**B**), and activity against preformed biofilms (**C**).

Besides *C. auris*, we were interested in assessing the inhibitory activity of our two main leading compounds against a number of *Candida* species that are also involved in human disease. Thus, we chose seven other species of *Candida* to test the compounds against in addition to *C. auris* 0390 (for comparison purposes). These included *C. albicans* strain SC5314, as well as clinical isolates of *C. dubliniensis*, *C. parapsilosis*, *C. tropicalis*, *C. glabrata* (obtained from the Fungus Testing Laboratory), and *C. krusei*, and *C. lusitaniae* (from the CDC panel). Again, we performed the three different methods for determining the activity against planktonic, inhibition of biofilm formation, and preformed biofilms. We found that miltefosine was effective at inhibiting growth, both under planktonic and biofilm growing conditions, of all *Candida* species tested, with the exception of *C. lusitaniae* (Figure 4A,B). Interestingly, in the case of preformed biofilms, miltefosine displayed the highest levels of activity against *C. auris*, although it was also active at higher concentrations against mature biofilms formed by *C. albicans*, *C. krusei*, *C. dubliniensis*, and *C. tropicalis*, with minimal activity against *C. glabrata* and virtually no activity against preformed biofilms of both *C. lusitaniae* and *C. parapsilosis* (Figure 4C).

**Figure 5:**
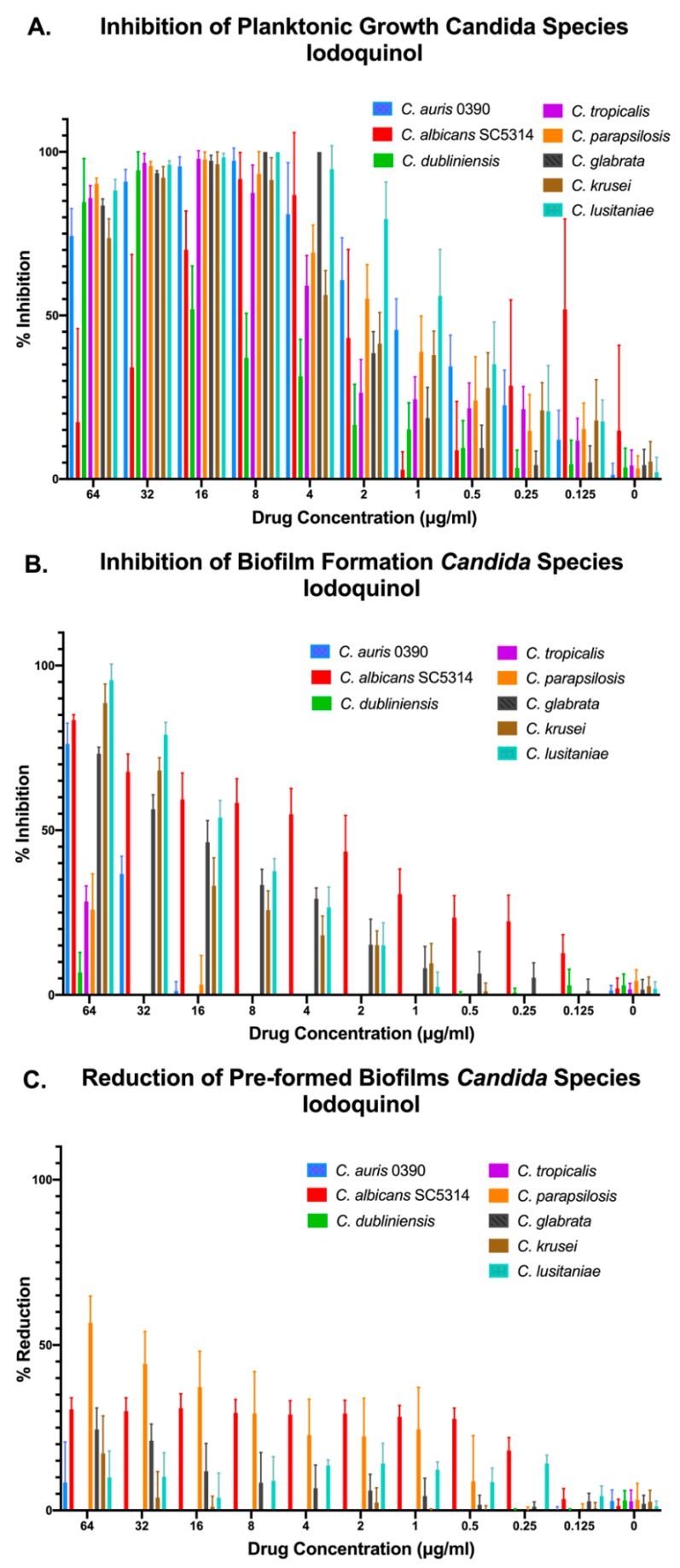
Activity of iodoquinol against representative strains from different *Candida* spp. under the three different growing conditions: inhibition of planktonic growth (**A**), inhibition of biofilm formation (**B**), and activity against preformed biofilms (**C**).

In the case of iodoquinol, results demonstrated its in vitro efficacy against most *Candida* species tested when growing under planktonic conditions, with the notable exception of *C. dubliniensis*. We note that in the case of *C. albicans* we observed a paradoxical effect at the highest concentrations of iodoquinol tested. Regarding the ability of iodoquinol to inhibit biofilm formation, we observed different degrees of inhibition depending on the species, with higher activity against *C. albicans* and moderate activity against *C. lusitaniae*, *C. krusei*, and *C. glabrata*; although we note that this activity was detected at relatively high concentrations (Figure 5A,B). As expected from initial results with *C. auris*, iodoquinol was mostly ineffective against preformed biofilms from all species tested (Figure 5C). 

## 4. Discussion

Since its first identification approximately 10 years ago, *C. auris* has emerged as a major cause of outbreaks of invasive candidiasis in healthcare facilities around the globe. These infections carry very high mortality rates, as high as 60% in some cases [9]. Given the paucity of clinically-used antifungal drugs, and since *C. auris* clinical isolates often exhibit high levels of resistance to multiple classes of antifungal drugs [29,33,44,45], there is dire and urgent need to identify compounds with activity against this devastating emerging pathogen. Repurposing (or repositioning) already existing drugs used to treat other ailments as antifungals represents a fast and economical alternative to bring drugs with novel antifungal activity from the bench to the bedside [34]. 

To this end, here we screened the Pathogen Box^®^ in search for drug-like molecules with inhibitory activity against *C. auris*. The Pathogen Box^®^ is a diverse library of compounds (400 drug-like molecules) originally assembled by MMV with the intention to accelerate the identification of drugs with in vitro activity against neglected diseases caused by parasites (eukaryotes), including Malaria, Leishmaniasis, and Chagas’s disease; worms, including *Schistosoma mansoni* and hookworm; and bacteria (prokaryotes; *Mycobacterium tuberculosis*). Some of the drugs in the library are effective against different classes/phyla and this encouraged us to search for activity against *C. auris*. Our group has previously reported on the identification of *C. albicans* biofilm inhibitors from the Pathogen Box^®^ [46], and the Kronstad group has also screened this same library for the identification of compounds with antifungal activity against both *C. albicans* and *Cryptococcus neoformans* [47]. As one of the factors contributing to the pathogenesis of *C. auris* is the formation of biofilms, and biofilms formed by this species are intrinsically resistant to all clinically-used antifungal agents [21,31,33], we initially decided to screen the library for inhibitors of *C. auris* growth under both planktonic and biofilm-growing conditions. Our initial screens resulted in the identification of 10 initial hit compounds with no primary antifungal designation that inhibited growth of *C. auris* by over 60% under either or both assay conditions; and results of subsequent confirmatory experiments confirmed the dose-response activity for four of them. Of these, miltefosine and iodoquinol represented the two leading repositionable compounds which we selected for a more in-depth characterization of their in vitro antifungal activity in follow-up experiments. 

Miltefosine (also referred as hexadecylphosphocholine, see Supplementary Figure S1) is an alkylphosphocholine drug originally developed as an anti-cancer drug, although its side effects (particularly liver toxicity) limited its utility for this purpose [48,49,50,51]. It was shown to possess activity against various parasites, most notably *Leishmania* spp. Miltefosine was approved for use in India in 2002 and more recently in the United States in 2014, and it is presently marketed by Zentaris GmbH (as Impavido) as a the first oral therapy of visceral and cutaneous leishmaniasis [52,53,54,55,56,57,58,59,60]. Depending on weight, Impavido is given at a dose of 50 mg 2–3 times a day for the treatment of leishmaniasis [61]. This treatment strategy allows for serum levels of up to 75.9 μg/mL in adults [61], and thus even the relatively high concentrations at which we observed activity against biofilms of *C. auris* and other *Candida* spp. should be achievable in patients. Miltefosine has dose-limiting gastrointestinal toxicity, but side effects are normally moderate in severity. It may also lead to hepatotoxicity and nephrotoxicity at very high doses. The drug was found to effect fertility in rats, although it has yet to be established whether humans can be similarly affected. In addition, it is not advised that this drug be taken during pregnancy or nursing as it can cause fetal mortality as well as adverse effects in infants [61]. Miltefosine has also been used successfully in a limited number of cases of the extremely rare highly lethal brain infection by the amoeba *Naegleria fowleri* and, in the United States, has orphan drug status for the treatment of other amebic infections [62]. Our group and others have previously described the antifungal activity of miltefosine [63,64,65,66,67,68]. Iodoquinol (also referred to as diiodohydroxyquinoline) is an halogenated quinoline derivative (see Supplementary Figure S1) that is used as an intestinal antiparasitic drug, mainly in the treatment of *Entamoeba histolytica* infections [69], apparently acting by chelation of ferrous ions essential for metabolism. Its poor intestinal absorption and side effects including on the central nervous system (i.e., seizures, and encephalopathy) and eye damage (optic atrophy leading to irreversible visual deterioration), have limited its systemic use [70]. In spite of these problems, it is used in topical creams to treat bacterial and fungal infections of the skin [71], and has recently been examined as a repositionable candidate for the treatment of multidrug resistant *Neisseria gonorrhoeae* [72]. We note that these two compounds were not identified before in previous screens against *C. albicans* and *C. neoformans* of the same Pathogen Box^®^ library [46,47].

Our in vitro tests confirmed the potent activity of iodoquinol against *C. auris* when growing planktonically, although the drug generally lacked biofilm inhibitory activity (Figure 3A). These findings are similar to those previously reported by our group for Ebselen, whose activity against planktonic *C. auris* was recently identified by our group after screening another repurposing library, the Prestwick library [73]; and most recently a different group also reported on the identification of several off-patent compounds with novel antifungal activity against planktonic *C. auris* after screening the same Prestwick library [74]. On the other hand, miltefosine displayed potent inhibitory activity against *C. auris* under both planktonic and biofilm-growing conditions (Figure 3B). However, perhaps the most remarkable finding was the activity of miltefosine, at concentrations that are achievable in patients [75], against preformed biofilms of *C. auris* (Figure 3B). This is particularly interesting since once formed these biofilms are notoriously difficult to treat due to their intrinsic resistance to all currently used antifungals [31,33]. These observations were not restricted to a single strain of *C. auris*, as all clinical isolates tested from the CDC panel showed similar in vitro susceptibility profiles for iodoquinol and miltefosine (Table 2). Moreover, both our leading repositionable candidates showed activity against a majority of *Candida* species (Figure 4 and Figure 5). 

In summary, we identified iodoquinol and miltefosine as our leading repositionable candidates in the Pathogen Box^®^ for the treatment of *C. auris* infections, for which there is a dire need to develop effective therapies. 

## Figures and Tables

**Table 1:** Identity and extent of inhibition of compounds identified as “hits” during primary screenings of the Pathogen Box^®^ in search for compounds with inhibitory activity against *C. auris* strain 0390 under planktonic and biofilm growing conditions.

Hit Compounds Inhibition of Planktonic Growth	% Inhibition of Planktonic Growth at 5 µM	% Inhibition of Planktonic Growth at 20 µM	Hit Compounds Inhibition of Biofilm Formation	% Inhibition of Biofilm Formation at 5 µM	% Inhibition of Biofilm Formation at 20 µM
**Amphotericin B**	93	89	Amphotericin B	97	99
**Posaconazole**	95	86	Posaconazole	72	78
**Miltefosine**	n/a	102	Miltefosine	n/a	98
**Iodoquinol**	86	98	Iodoquinol	n/a	n/a
**MMV676162**	n/a	65	MMV676162	n/a	n/a
**Pentamidine**	n/a	84	Pentamidine	61	n/a
**MMV102872**	n/a	88	MMV102872	n/a	n/a
**MMV688852**	n/a	70	MMV688852	n/a	n/a
**MMV687775**	n/a	61	MMV687775	n/a	n/a
**MMV688417**	n/a	60	MMV688417	n/a	n/a
**MMV659010**	62	n/a	MMV659010	n/a	n/a
**MMV688938**	n/a	62	MMV688938	n/a	n/a

n/a indicates the inhibitory values below the target value of 60% inhibition.

**Table 2:** Calculated IC_50_ values for the two main repositionable compounds miltefosine and iodoquinol against different *C. auris* strains from the CDC panel, under the three different growing conditions: inhibition of planktonic growth, inhibition of biofilm formation, and activity against preformed biofilms. Values are in μg/mL.

*C. auris* Strain	IC_50_ of Inhibition of Planktonic Growth		IC_50_ of Inhibition of Biofilm Formation		IC_50_ of Reduction of Pre-Formed Biofilms	
	Miltefosine	Iodoquinol	Miltefosine	Iodoquinol	Miltefosine	Iodoquinol
**0381**	0.9237	0.7959	1.158	24.47	9.144	>64
**0382**	2.472	0.4489	4.988	27.79	14.69	>64
**0383**	1.379	1.571	1.702	52.12	9.449	>64
**0384**	1.404	2.006	2.645	56.02	9.214	>64
**0385**	2.149	1.565	4.656	33.60	14.84	38.58
**0386**	2.152	0.8520	4.538	25.88	14.59	49.11
**0387**	2.265	0.7733	5.160	14.50	13.91	>64
**0388**	2.129	0.4545	5.374	25.37	20.98	>64
**0389**	2.139	0.6701	6.049	9.159	13.62	>64
**0390**	1.984	0.2972	3.932	43.40	13.91	>64

## Supplementary Materials

The following are available online at https://www.mdpi.com/2309-608X/5/4/92/s1, Figure S1: Chemical structures of miltefosine and iodoquinol.
