# Supplementary material for: Repositionable Compounds with Antifungal Activity against Multidrug Resistant Candida auris Identified in the Medicines for Malaria Venture’s Pathogen Box
Source: J Fungi (Basel). 2019 Oct 1;5(4):92. doi: 10.3390/jof5040092 (PMC6958377; doi:10.3390/jof5040092)
Supplement: Supplementary file 1 [file jof-05-00092-s001.pdf]

A. Miltefosine

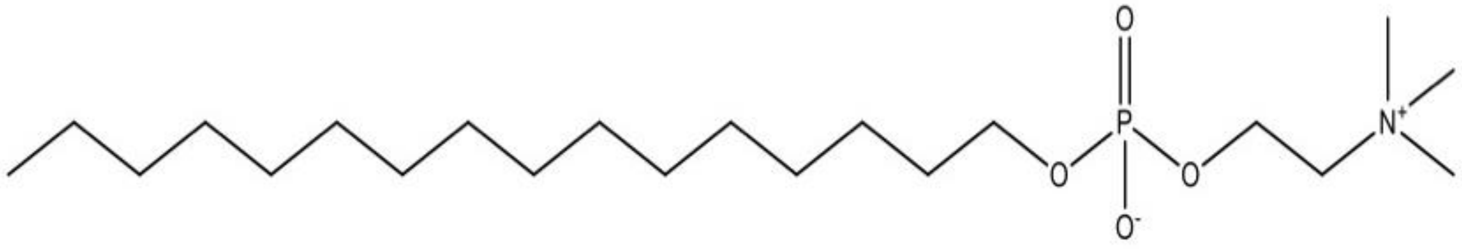

B. Iodoquinol

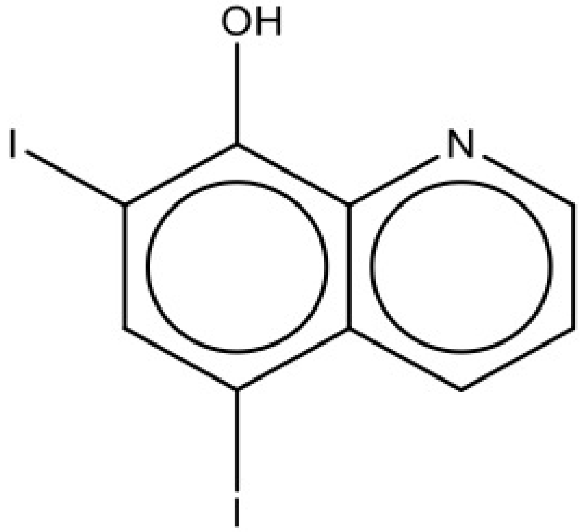

Supplementary Figure 1. Chemical structures of miltefosine (A) and iodoquinol (B)
